# The updated JSPGHAN guidelines for the management of *Helicobacter pylori* infection in childhood

**DOI:** 10.1111/ped.14388

**Published:** 2020-12-22

**Authors:** Seiichi Kato, Toshiaki Shimizu, Shigeru Toyoda, Benjamin D. Gold, Shinobu Ida, Takashi Ishige, Shigeru Fujimura, Shigeru Kamiya, Mutsuko Konno, Kentaro Kuwabara, Kosuke Ushijima, Norikazu Yoshimura, Yoshiko Nakayama, the Japanese Society for Pediatric Gastroenterology, Hepatology and Nutrition

**Affiliations:** ^1^ Kato Children’s Clinic Natori Japan; ^2^ Department of Infectious diseases Kyorin University School of Medicine Tokyo Japan; ^3^ Department of Pediatrics and Adolescent Medicine Juntendo University Graduate School of Medicine Tokyo Japan; ^4^ Nojiri Children and Family Clinic Fujinomiya Japan; ^5^ Children’s Center for Digestive Healthcare, LLC Atlanta GA USA; ^6^ Department of Pediatric Gastroenterology and Endocrinology Osaka Women’s and Children’s Hospital Osaka Japan; ^7^ Department of Pediatrics Gunma University Graduate School of Medicine Maebashi Japan; ^8^ Division of Clinical Infectious Diseases & Chemotherapy Tohoku Medical and Pharmaceutical University Graduate School of Pharmaceutical Sciences Sendai Japan; ^9^ Department of Pediatrics Sapporo Kosei General Hospital Sapporo Japan; ^10^ Department of Pediatrics Hiroshima City Hiroshima Citizens Hospital Hiroshima Japan; ^11^ Department of Pediatrics and Child Health Kurume University School of Medicine Kurume Japan; ^12^ Yoshimura Medical Clinic Osaka Japan; ^13^ Department of Pediatrics Shinshu University School of Medicine Matsumoto Japan

**Keywords:** *Helicobacter pylori*, child, eradication therapy, gastric cancer, antibiotic resistance

## Abstract

The Japan Pediatric *Helicobacter pylori* Study Group published the first guidelines on childhood *H. pylori* infection in 1997. They were later revised by the Japanese Society for Pediatric Gastroenterology, Hepatology and Nutrition (JSPGHAN). The *H. pylori* eradication rates, when employing triple therapy with amoxicillin and clarithromycin, currently recommended as the first‐line therapy of *H. pylori* infection in Japan, have substantially decreased, creating an important clinical problem worldwide. In Japanese adults, the “test‐and‐treat” strategy for *H. pylori* infection is under consideration as an approach for gastric cancer prevention. However, the combined North American and European pediatric guidelines have rejected such a strategy for asymptomatic children. As risk for gastric cancer development is high in Japan, determining whether the “test‐and‐treat” strategy can be recommended in children has become an urgent matter. Accordingly, the JSPGHAN has produced a second revision of the *H. pylori* guidelines, which includes discussion about the issues mentioned above. They consist of 19 clinical questions and 34 statements. An *H. pylori* culture from gastric biopsies is recommended, not only as a diagnostic test for active infection but for antimicrobial susceptibility testing to optimize eradication therapy. Based upon antimicrobial susceptibility testing of *H. pylori* strains (especially involving clarithromycin), an eradication regimen including use of the antibiotics to which *H. pylori* is susceptible is recommended as the first‐line therapy against *H. pylori*‐associated diseases. The guidelines recommend against a “test‐and‐treat” strategy for *H. pylori* infection for asymptomatic children to protect against the development of gastric cancer because there has been no evidence supporting this strategy.

## Introduction

Gastric colonization with *Helicobacter pylori* in the human stomach persists in some individuals for a long time and is closely associated with the development of gastritis, gastric or duodenal ulcers, or extra‐gastrointestinal diseases such as iron deficiency anemia (IDA) in childhood. It is thought that long‐term persistent infection with the microorganisms leads to the development of gastric cancer – in particular, adenocarcinoma, especially the intestinal type – in a subset of the infected adults. In 1997, the Japan Pediatric *H. pylori* Study Group published the first practice guidelines for *H. pylori* infection in childhood.^1^ In 2005, the guidelines were revised by the Japanese Society for Pediatric Gastroenterology, Hepatology and Nutrition (JSPGHAN).^2^

In Japan, proton‐pump inhibitor‐based triple therapy with amoxicillin and clarithromycin (PAC regimen) is recommended as the first‐line therapy for *H. pylori*‐associated diseases in children.^2^ However, eradication rates of PAC regimen have decreased year by year, especially because *H. pylori* strains are increasingly resistant to clarithromycin. We are now forced to take prompt measures to address and account for the increasing antibiotic resistance of *H. pylori*, especially clarithromycin.

In the adult Japanese population, the so called “test‐and‐treat” strategy for *H. pylori* infection is now under consideration for gastric cancer prevention. On the other hand, the majority of children with *H. pylori* infection remain asymptomatic, although a percentage of the infected children do develop *H. pylori‐*associated diseases. The previous pediatric *H. pylori* guidelines in Japan^2^ did not recommend a “test‐and‐treat” strategy for *H. pylori* infection in asymptomatic children. Recently, North American Society for Pediatric Gastroenterology, Hepatology and Nutrition (NASPGHAN) and European Societies for Paediatric Gastroenterology, Hepatology and Nutrition (ESPGHAN) have updated the joint *H. pylori* guidelines.^3^ The joint guidelines have recommended against a “test‐and‐treat” strategy for *H. pylori* infection in children because, since the previously published joint transatlantic guidelines in 2011, there has been no evidence demonstrating improved outcomes in *H. pylori*‐infected children to support such strategy. However, because Japan is a country where there is a high risk of gastric cancer development, determining whether such a “test‐and‐treat” strategy can be recommended in asymptomatic children has become an urgent issue. Accordingly, the JSPGHAN has produced a second revision of the guidelines on the management of childhood *H. pylori* infection as of October 2018, mainly focusing on appropriateness of a “test‐and‐treat” strategy and measures to address the decreasing success rate of eradication therapies.^4^ The present guidelines are an English version of the revised guidelines.^4^ In the present English version, the introduction, the methods, and the comments for the statements have been shortened.

At present, JSPGHAN has provided the guidelines based upon the best available and most recently published evidence. However, responsibility for the management of *H. pylori* infection in children, including the choice of diagnostic tests, eradication therapy, and its clinical effects, rests with physicians who perform such practices but not with JSPGHAN.

## Methods

### Purpose and subjects

Based upon new evidence about childhood *H. pylori* infection, the JSPGHAN agreed to update the guidelines on the management of *H. pylori*‐related diseases in Japanese children,^2^ including an attempt to offer recommendations as to how asymptomatic children should be treated. The present guidelines comprise clinical questions and corresponding statements and comments. The most recent guidelines apply to children aged 15 years or younger, who include junior high school students in Japan. The *H. pylori*‐related disorders discussed in the guidelines include histological chronic gastritis, peptic ulcer disease, and extra‐gastrointestinal diseases such as IDA or idiopathic (immune) thrombocytopenic purpura (ITP).

### Guideline Committee and Literature Review

In the Japanese version, the guideline committee consisted of 12 experts on childhood *H. pylori* infection, including pediatric gastroenterologists and microbiologists, who then voted on each of the statements. B. D. Gold, who has participated on the majority of the North American, Canadian and Joint NASPGHAN‐ESPGHAN guidelines, worked on writing the English version but did not vote in the Japanese version. The guidelines were revised based on the guideline development process proposed by the Medical Information Network Distribution Service (Minds).^5^ Twelve members of the committee were divided into three groups, namely (i) review of the *H. pylori*‐associated diseases including the management of asymptomatic children, (ii) diagnostic methods, and (iii) treatment, particularly paying attention to addressing the clinically important issue of antimicrobial resistance. Several committee members participated in more than one group. In each group, the members wrote the first draft of clinical questions and the corresponding statements and comments. The systematic review team (S.K., N.H., and T.I. in the acknowledgments) was organized in the guideline committee. The review team performed a systematic review of all peer‐reviewed academic manuscripts, including guidelines and systematic reviews, published until October 2016. With regard to English manuscripts, MEDLINE and the Cochrane Library were searched using key words and terms, including *H. pylori,* extracted from “clinical questions.” The review team also searched the International Guideline Library and National Guideline Clearinghouse (NGC) to pick up important overseas guidelines. Igaku Chuo Zasshi, Japan’s largest medical‐literature database, was used to search peer‐reviewed Japanese manuscripts. The present guidelines also included several important overseas guidelines or systematic reviews on childhood *H. pylori* infection, which have been published since November 2016.

### Evidence Level

In each statement, the evidence levels were classified into A (high), B (moderate), C (low), and D (very low), based on quality of evidence. In principle, systematic review, meta‐analyses, and randomized controlled trials were placed in level A for quality of evidence, non‐randomized, cohort and case‐control studies were placed in level B or C, and case series in level D.

### Consensus Process and Strength of Recommendation

A modified Delphi technique was used to develop a consensus statement based on critical evaluation of the evidence on each statement, anonymous votes, discussion and modification of the statement, and repeated anonymous votes. Votes for each statement were repeated a maximum of three times to arrive at an agreement. In each statement, a vote was conducted using a four‐point scale: agree strongly (A+), just agree (A), just disagree (D), and disagree strongly (D+). On each statement, consensus was defined as agreement (the sum of A+ and A) of 70% or higher of the voting members. If disagreement (the sum of D+ and D) was 70% or higher in the end, the statement was withdrawn. The sum of A+ and A was also described as “Agreement (%).” In the statement which was not applicable to consensus statement, “Not applicable” was mentioned on Agreement (%). According to the process of evidence evaluation and the quality of evidence for each statement, the committee’s recommendations were graded as either *Strong* or *Weak*. Voting on each statement was repeated a maximum of three times until either a rating of *Strong* or *Weak* had been reached by the Committee. Ratings of *Strong* or *Weak* were obtained when ≥70% of voting members reached agreement on the statement. When the strength of recommendation did not reach agreement with the final vote, the strength was described as *Not determined*. Overall discussion on the guidelines was done at a face‐to‐face meeting in Tokyo.

## *H. pylori*‐associated diseases

**[Clinical Question 1]**

**Are eradication therapies recommended for all children with *H. pylori*‐associated diseases?**

**Statement 1: Eradication therapy should be considered for children, 5 years of age or more, determined to be infected with *H. pylori* by a test for active infection, taking account possible re‐infection.**

Strength of recommendation: Weak. Evidence level: C. Agreement: 92%.

***Comments***

In children aged 5 years or older, rates of *H. pylori* re‐infection are low at 2.0–2.4% per year.^6^, ^7^ It has therefore been suggested that eradication therapy should be recommended in children aged 5 years or older.^8^, ^9^ Konno *et al*. reported that mother‐to‐child transmission of *H. pylori* can occur in children under 5 years old in Japan.^9^ Considering these facts, in principle we recommend eradication therapy in children aged 5 years or older. However, eradication therapy should be considered in children under 5 years in whom the therapy is clinically indicated due to the disease or condition requiring a work up that results in the diagnosis of *H. pylori* infection including peptic ulcer diseases with stenotic lesion, perforation or recurrent hemorrhage, or MALT (mucosa‐associated lymphoid tissue lymphoma). It is important to note that it has also been reported that rates of re‐infection are low in children aged 7 years or older^10^ and that there does not appear to be an association between re‐infection rate and the age which eradication therapy is achieved.^11^, ^12^ Further investigation is therefore required about the specific age which is most valid for eradication therapy.

**[Clinical Question 2]**

**Are eradication therapies recommended for *H. pylori*‐infected children with peptic ulcer disease?**

**Statement 2: We recommend eradication therapy for *H. pylori*‐infected children with gastric and/or duodenal ulcers.**

Strength of recommendation: Strong. Evidence level: A. Agreement: 100%.

***Comments***

In meta‐analyses in adults with *H. pylori*‐associated gastric or duodenal ulcers,^13^, ^14^ patients with successful eradication significantly increased healing rates and decreased relapse rates compared with those with failed eradication or without eradication therapy.^13^, ^14^ In a randomized controlled trial,^15^ *H. pylori* eradication has accelerated rates of ulcer healing in children with *H. pylori*‐associated peptic ulcer disease. In a cohort study of children with duodenal ulcers,^16^ ulcer recurrence rates after *H. pylori* were successfully eradicated were low with rates of 9% per year at, on average, 2 years’ follow up.

In Japanese children, the *H. pylori* prevalence was 83% in duodenal ulcer and 44% in gastric ulcers, respectively.^17^ In a follow‐up study, seven out of 27 pediatric patients with peptic ulcer disease showed recurrence of the disease and all were more than 10 years old.^18^ In Taiwanese children with peptic ulcer disease, the first cause was *H. pylori,* which was involved in a half of the patients, and the second was non‐steroidal anti‐inflammatory drugs (NSAIDs).^19^ The joint ESPGHAN/NASPGHAN guidelines have recommended tests and treatment in children with gastric or duodenal ulcer disease.^3^

In acute gastric ulcers, which were clinically categorized as acute gastric mucosal lesion (AGML) in Japan, one out of six cases showed chronic persistent *H. pylori* infection.^20^ *H. pylori* was histologically found in gastric biopsy specimens of 75% of children with AGML.^21^ *H. pylori* testing should be considered in children with AGML, but eradication therapy should be undertaken when chronic persistent active infection is confirmed.

**[Clinical Question 3]**

**What kinds of clinical conditions in which *H. pyolri* infection has been determined are appropriate for eradication therapies when upper gastrointestinal endoscopy was performed for abdominal symptoms?**

**Statement 3‐1: We recommend consideration of eradication therapy for *H. pylori*‐infected children who underwent diagnostic upper gastrointestinal endoscopy for abdominal symptoms.**

Strength of recommendation: Weak. Evidence level: D. Agreement: 100%.

***Comments***

Eradication therapy is thought to be ineffective for the resolution of abdominal symptoms of pediatric patients without peptic ulcer disease.^3^, ^22^, ^23^, ^24^ A meta‐analysis^22^ suggests that prevalence of abdominal symptoms does not differ between *H. pylori*‐positive and ‐negative patients. Bode *et al*.^23^ demonstrated an association between prevalence of abdominal pain or vomiting and positivity of urea breath test, and Dore *et al*.^24^ showed that there was no association between nausea / vomiting or diarrhea and serum *H. pylori* antibody titer. The updated NASPGHAN/ESPGHAN guidelines recommended endoscopic examination as well as *H. pylori* testing for diagnosing pediatric patients with alarm signs or symptoms (“alarm features”) associated with abdominal symptoms.^3^ The guidelines also recommended against *H. pylori* testing for patients diagnosed with functional abdominal pain.

On recurrent abdominal pain (RAP), some studies suggest that there is no association between *H. pylori* infection and the symptoms.^3^, ^23^ Despite the weakness of antibody testing as a reflection of active infection, Bode *et al*.^23^ and Tindberg *et al*.^25^ reported no association with serum *H. pylori* antibody titers and RAP symptoms. A retrospective cohort study reported significant improvement of RAP symptoms in patients with successful eradication compared to those with failed eradication.^26^ However, prospective cohort studies^27^, ^28^ and a randomized controlled study^29^ have reported no significant differences in symptomatic improvement of RAP between both groups.

Based upon this evidence it appears that *H. pylori* eradication does not improve the abdominal symptoms of RAP in children with the infection. However, the Maastricht III consensus report recommended performing upper gastrointestinal endoscopy and *H. pylori* testing, as well as initiating eradication therapy for the infected individuals with symptoms on the upper abdomen, particularly if other causes are ruled out.^30^

Although eradication therapy is considered for patients in whom *H. pylori* infection is confirmed and evident endoscopic findings are shown, physicians should discuss with the patients and family the advantages and disadvantages of eradication therapy, taking into consideration the potential risk and benefits of treatment.

**Statement 3‐2: We recommend consideration of eradication therapy for *H. pylori*‐infected children with histological evidence of chronic gastritis, in the absence of ulcers, to improve mucosal inflammation in the stomach.**

Strength of recommendation: Weak. Evidence level: B. Agreement: 100%.

**Statement 3‐3: We recommend eradication therapy for all *H. pylori*‐infected children in whom gastric atrophy (with or without intestinal metaplasia) is histologically shown.**

Strength of recommendation: Not determined. Evidence level: D. Agreement: 100%.

***Comments:*** common to Statements 3‐2 and ‐3.

Eradication therapy for chronic *H. pylori* gastritis is strongly recommended in adult patients because, with clearance of the infection, there is an improvement in mucosal atrophy, reduced development of intestinal metaplasia, and a prophylactic effect against gastric cancer development can be expected.^31^, ^32^ As in adults, *H. pylori*‐infected children show frequently histologic chronic gastritis and, rarely, have been demonstrated to also have gastric mucosal atrophy.^33^, ^34^ In children with histologic chronic gastritis, however, it remains to be definitively determined whether *H. pylori* eradication can improve mucosal inflammation and the associated symptoms.

It is reported that body‐mass index, and serum ghrelin and leptin concentrations significantly decreased after *H. pylori* eradication in 30 pediatric patients with *H. pylori* chronic gastritis.^35^ In a randomized control study by Buonavolonta *et al.,* ^36^ endoscopic findings and histological score of chronic inflammation significantly improved at 1 year follow up after *H. pylori* eradication in comparison with the group without eradication therapy. Several cohort studies also suggest the effectiveness of eradication therapy on histological inflammation in pediatric patients with *H. pylori* chronic gastritis.^34^, ^37^ Considering these facts, histological improvement may be expected after *H. pylori* is eradicated in the infected children with chronic gastritis.

**[Clinical Question 4]**

**Are eradication therapies recommended for *H. pylori*‐infected children with gastric mucosa‐associated lymphoid tissue (MALT) lymphoma?**

**Statement 4: We recommend eradication therapy for *H. pylori*‐infected children with gastric MALT lymphoma.**

Strength of recommendation: Strong. Evidence level: B. Agreement: 100%.

***Comments***

There is only one review^8^ and three case reports^38^, ^39^, ^40^ about *H. pylori* eradication therapies for MALT lymphoma in children. In a 14‐year‐old American girl with *H. pylori*‐positive gastritis and low‐grade MALT lymphoma, the lymphoproliferative lesion was improved with the eradication therapy and no recurrence of the lesion was detected at 7 year follow up.^38^ In Japanese boys aged 6 years and 14 years with gastric MALT lymphoma in an immunosuppressed state, the lymphoma disappeared in both cases after *H. pylori* was eradicated and no recurrence was demonstrated at 3 year and 10 year follow up.^39^ In an 8‐year‐old Saudi Arabian boy with IDA and *H. pylori*‐positive gastritis, high‐grade B cell lymphoma was detected 3 months after completion of eradication therapy but the lymphoma lesion completely disappeared with continuing eradication therapy.^40^ We recommend consideration of eradication therapy in children with gastric MALT lymphoma, when *H. pylori* infection is confirmed.

**[Clinical Question 5]**

**Are eradication therapies recommended for *H. pylori*‐infected children with protein‐losing gastro‐enteropathy?**

**Statement 5: We recommend considering eradication therapies for *H. pylori*‐infected children with protein‐losing gastro‐enteropathy, if other etiologies for the gastrointestinal protein loss are not found.**

Strength of recommendation: Weak. Evidence level: C. Agreement: 100%.

***Comments***

There are no systematic reviews or randomized controlled trials with regard to this clinical question. On the other hand, many case series have reported the effectiveness of *H. pylori* eradication therapy for children with protein‐losing gastro‐enteropathy.^41^, ^42^, ^43^, ^44^, ^45^, ^46^ Although the pathogenesis remains to be determined, causes such as cytomegalovirus infection, Menetrier's disease, eosinophilic gastroenteritis and intestinal lymphangiectasia should be considered and evaluated. When such causes are ruled out, eradication therapy may be considered.

**[Clinical Question 6]**

**Are eradication therapies recommended for *H. pylori*‐infected children with iron deficiency anemia (IDA)?**

**Statement 6: We recommend eradication therapy for *H. pylori*‐infected children with IDA when the iron deficiency is recurrent or refractory to iron supplement therapy.**

Strength of recommendation: Strong. Evidence level: A. Agreement: 100%.

***Comments***

The pathogenesis of IDA caused by *H. pylori* infection in adults has been reported to be contributed to in part by hypoacidity due to gastric mucosal atrophy and a decrease in the ascorbic acid concentration in the stomach,^47^ or an increased level of serum hepcidin^48^ associated with impaired iron absorption from the upper part of the small intestine. However, in children with *H. pylori* chronic gastritis, severe gastric mucosal atrophy is rare,^49^ and gastric acid secretion in these children does not differ from those without *H. pylori* infection.^50^ On the other hand, with regard to bacterial factors related to IDA caused by *H. pylori*, differences in the ability of iron uptake among *H. pylori* strains^51^ have been shown and these observed differences are likely due to genetic polymorphisms in napA gene involved in iron acquisition.^52^, ^53^

In general, cases with IDA caused by *H. pylori* infection are frequently refractory to iron supplement therapy or give rise to recurrent episodes, and eradication therapy without iron supplementation has resulted in cure of the disease.^54^, ^55^ *H. pylori‐*associated IDA frequently occurs in adolescence, when iron demand increases with growth spurts and sports activities.^54^, ^55^ Meta‐analyses showed that a risk of iron deficiency and IDA is higher in individuals with *H. pylori* infection than in those without the infection.^56^, ^57^ In IDA caused by an *H. pylori* infection, the improvement of blood ferritin and hemoglobin levels was more frequently shown in groups with iron supplement therapy alone than with a combination of *H. pylori* eradication and iron supplement.^56^, ^57^ The Maastricht V/ Florence Consensus Report^58^ recommends eradication therapy for *H. pylori*‐infected persons with IDA. Furthermore, the updated ESPGHAN / NASPGHAN guidelines suggest *H. pylori* testing in children with refractory IDA in which other causes have been ruled out.^3^ Eradication therapy should be considered in children who have recurrent or refractory IDA to iron supplementation and in whom an active *H. pylori* infection has been determined.

**[Clinical Question 7]**

**Are eradication therapies recommended for *H. pylori*‐infected children with chronic idiopathic (immune) thrombocytopenic purpura (ITP)?**

**Statement 7: We recommend eradication therapy for *H. pylori*‐infected children with chronic ITP as the first‐line therapy.**

Strength of recommendation: Strong. Evidence level: B. Agreement: 100%.

***Comments***

In adults with ITP, it was reported that platelet counts increased in 60% of the patients with successful *H. pylori* eradication.^59^ The Japanese Society of Hematology published a clinical guide that recommends *H. pylori* testing for patients with chronic ITP and eradication therapy for the *H. pylori*‐infected patients prior to the use of corticosteroid hormones as a first‐line therapy.^60^ Childhood ITP is mainly the acute type and 85% of the patients recover within 6 months after onset. Although the positivity rate of *H. pylori* infection in children with chronic ITP is lower than in adults with the disease, the updated ESPGHAN / NASPGHAN guidelines suggest non‐invasive *H. pylori* testing for active infection when investigating causes of chronic ITP.^3^ *H. pylori* eradication is effective in approximately 40% of cases with chronic ITP, although there are only reports for a small number of cases.^61^ In patients with chronic ITP, eradication therapy seldom causes a deterioration of the clinical course and adverse effects.^62^, ^63^, ^64^ Eradication therapy should therefore be considered as the first‐line treatment for pediatric patients with chronic ITP, if *H. pylori* infection is confirmed.^60^, ^65^, ^66^ In children with chronic ITP in whom thrombocytopenia continues regardless of standard therapies according to Japanese clinical practice guidelines for the management of pediatric ITP,^67^ *H. pylori* testing should be performed without endoscopy and, if the infection is confirmed, eradication therapy can be attempted.

**[Clinical Question 8]**

**Are eradication therapies recommended for *H. pylori*‐infected children with urticaria?**

**Statement 8: We do not recommend eradication therapies for *H. pylori*‐infected children with chronic idiopathic urticaria.**

Strength of recommendation: Not determined. Evidence level: C. Agreement: Not reached.

***Comment***

The cause of chronic idiopathic urticaria (CIU) is unknown. In case‐control studies,^68^, ^69^, ^70^, ^71^ case series,^72^, ^73^ and pediatric case reports,^74^ it is reported that *H. pylori* eradication was effective in CIU with the infection, although these studies were primarily carried out in adult patients. In a case‐control study in Japan,^69^ rates of complete or partial remission of the CIU were significantly higher in the patients with successful *H. pylori* eradication than in those with eradication failure. In a Japanese case series including pediatric patients,^72^ CIU patients with successful *H. pylori* eradication showed a significantly higher rate of the complete or partial remission of their chronic urticaria in comparison with those with eradication failure. A more recent pediatric case series^75^ demonstrated that CIU showed high prevalence rates of *H. pylori* infection with the rate of 31.2% and the symptoms disappeared after successful eradication in all the infected patients. In this single‐center study, all of the *H. pylori*‐positive patients were 8 years of age or older. Thus, although the underlying pathophysiology or etiologic mechanism remains unclear, there is a possibility that *H. pylori*‐positive CIU is improved or healed by eradication therapy. Eradication therapy might be a choice of treatment in schoolchildren with CIU, although at this time, supporting evidence is not sufficient to make that recommendation.

In a pediatric case in Japan,^76^ it was reported that the symptoms of atopic dermatitis almost disappeared after *H. pylori* was eradicated. However, meta‐analyses have suggested an inverse association between *H. pylori* prevalence and development of allergic diseases including atopic dermatitis.^77^, ^78^ In an experimental study in mice, tolerization with *H. pylori* extract prevents airway hyper‐responsiveness, bronchoalveolar eosinophilia, and Th2 cytokine production.^79^ Further studies are needed to determine if there is a clear benefit from eradication therapy for atopic dermatitis or CIU, particularly because of epidemiological and animal model data that suggest that *H. pylori* may improve allergic symptoms / processes and that there is an inverse association between *H. pylori* infection and development of allergic or atopic diseases.

**[Clinical Question 9]**

**Are eradication therapies recommended for asymptomatic children with *H. pylori* infection?**

**Statement 9‐1: We recommend against a “test‐and‐treat” strategy for *H. pylori* infection for asymptomatic children to protect gastric cancer development.**

Strength of recommendation: Not determined. Evidence level: C. Agreement: 100%.

***Comment***

*H. pylori* eradication reduces gastric cancer risk in the adult population.^80^, ^81^, ^82^, ^83^, ^84^ In Japanese adults, a test‐and‐treat strategy for gastric cancer prevention is under discussion. In some cities or towns in Japan, a “test‐and‐treat strategy” for junior high school children is performed at municipality level. On the other hand, North American and European pediatric guidelines^3^, ^85^ have recommended against a “test‐and‐treat” strategy for *H. pylori* infection in asymptomatic children.

It seems difficult or even impossible to determine whether a “test‐and‐treat” strategy for *H. pylori* infection in childhood would reduce the risk of gastric cancer development during adulthood.^86^ In a pediatric review,^86^ severe gastric atrophy and intestinal metaplasia, which are known as risk factors of intestinal type of gastric cancer, were reported to be generally rare. The authors indicated the lack of validated criteria on severity assessment of gastric histology in pediatric studies. A study of Japanese children (*n* = 196), based on the updated Sydney System,^49^ reported as follows: (i) *H. pylori* does not appear to induce severe atrophy in the gastric body, whereas the microorganisms can infrequently induce moderate atrophy in the antrum; (ii) intestinal metaplasia rarely develops, whereas the lesion is also detected in children without *H. pylori* infection; (iii) *H. pylori‐*associated gastritis is antrum‐predominant. Uemura *et al*. have indicated that in Japanese adults, risk factors of intestinal‐type gastric cancer are severe gastric atrophy and corpus‐predominant *H. pylori* gastritis,^83^ suggesting that risk of intestinal‐type gastric cancer is low in children. A Japanese pediatric study^49^ was re‐analyzed according to the Operative Link on Gastritis Assessment staging system. There were no cases with stage IV, which is thought to be serious precancerous stage.^87^ Gastric acid secretion does not differ between Japanese children with and without *H. pylori* gastritis, showing that the infected children have no significant gastric atrophy.^50^ In Korea, a country with a high risk of gastric cancer, like Japan, significant atrophy in children has not been reported.^88^ These findings suggest that children with *H. pylori* infection do not appear to have an increased gastric cancer risk during childhood.

On the other hand, risk of diffuse‐type gastric cancer may be associated with mild to moderate gastric atrophy.^83^ However, there are no reports about the association of this histopathological phenotype in children. Nodular gastritis is a typical *H. pylori‐*associated chronic gastritis in childhood,^17^ but the population‐based epidemiology of this endoscopically detectable lesion and phenotype has not been characterized, and it is unknown whether childhood nodular gastritis could lead to development of diffuse‐type gastric cancer in adulthood.

With regard to gastric cancer prevention with *H. pylori* eradication, Asaka *et al*. estimated that the cancer development can be prevented in 93–98% of Japanese adults aged 40–49 years and in almost 100% of those aged <40 years.^89^ A “test‐and‐treat” strategy for *H. pylori* infection for adults would therefore protect against gastric cancer development effectively. However, a “test‐and‐treat” strategy, as performed in some cities or towns in Japan, is not recommended for children. On the other hand, the clinicians should take measures to reduce families’ anxiety about gastric cancer development, and because Japan is a high‐risk country, especially when children have family history of gastric cancer (refer to CQ9‐2), validation of testing and then treatment options should be discussed with the family. On the other hand, meta‐analyses have suggested that *H. pylori* infection may show inhibitory effects for childhood allergic diseases or at least be a surrogate marker for increased hygiene and thereby an increased atopic disease.^77^, ^78^ We should also consider that a “test‐and‐treat” strategy might produce negative effects in children. In any case, the clinical practice of *H. pylori* testing and eradication therapy in asymptomatic children should be left to the discretion of the treating physician and the families.

**Statement 9‐2: We recommend consideration of eradication therapies for children who have a family history of gastric cancer in their first‐ or second‐degree relatives and in whom active *H. pylori* infection has been found.**

Strength of recommendation: Weak. Evidence level: B. Agreement: 100%.

***Comment***

Case‐control studies outside of Japan^90^, ^91^, ^92^ have reported that risk of gastric cancer development is high in the first‐degree relatives of the index cases. Canadian pediatric guidelines proposed *H. pylori* testing in children who have a family history of gastric cancer, whereas the guidelines recommended against the testing for preventing gastric cancer in asymptomatic children.^85^ Considering that Japan is a country with a high risk of gastric cancer, we have proposed a “test‐and‐treat” strategy for *H. pylori* infection in children who are the first‐ or second‐degree relatives of the index case with gastric cancer, if their guardians are anxious about the future development of gastric cancer and request such practices.

**Statement 9‐3: We recommend against a “test‐and‐treat” strategy for asymptomatic children living in the household of an *H. pylori*‐infected adult who received eradication therapy to prevent re‐infection in that adult.**

Strength of recommendation: Weak. Evidence level: B. Agreement: 100%.

***Comment***

Molecular fingerprinting techniques^93^ demonstrated that the recurrence of *H. pylori* within 12 months after eradication therapy is mainly recrudescence with the same strains before and after the therapy but not real recurrence or re‐infection with the different strains. Recent meta‐analyses^93^, ^94^ and a US cohort study,^95^ in which recrudescence cases were excluded, showed that recurrence rates of *H. pylori* infection after successful eradication are very low. In Japan, recurrence rates of *H. pylori* infection after eradication success were reported to be low with 0.22–2.0% per year in adults^96^, ^97^, ^98^ and with 2.4% per year in children.^6^ Similarly, studies in Korea,^99^ Brazil^100^ and China^101^ have all shown that recurrence rates after eradication therapy are very low.

On recurrence of *H. pylori* infection after successful eradication, the spouse and children of persons who received eradication therapy are not considered risk factors for reinfection and the recurrence rates are very low even if other family members have the infection.^102^, ^103^ There is no evidence supporting a “test‐and‐treat” strategy for *H. pylori* infection for asymptomatic children to prevent re‐infection in the adult who received the eradication therapy. Therefore, re‐examination of *H. pylori* testing – i.e. accuracy of the test, confounding factors, e.g. on a proton pump inhibitors (PPIs) – is adequate for investigating possible recurrence of the infection after successful eradication.

## Diagnostic methods

**[Clinical Question 10]**

**Which tests are recommended for the diagnosis of *H. pylori* infection in children?**

### A) Diagnostic tests using endoscopic biopsy specimens

**Statement 10‐1‐1: We recommend considering the performance of a rapid urease test directly on gastric biopsies to determine presence / absence of *H*** *.* ***pylori* as a diagnostic test for active infection.**

Strength of recommendation: Weak. Evidence level: C. Agreement: 100%.

***Comments***

Diagnostic tests to detect *H. pylori* are divided into invasive “point” examinations using endoscopic biopsy specimens and non‐invasive “surface” examinations without biopsy specimens. The sensitivity and specificity of each diagnostic test are shown in Table 1. Diagnostic tests, except for antibody tests for *H. pylori,* should be performed at least 2 to 4 weeks after stopping antibiotics or PPIs. The rapid urease test (RUT) has been regarded as the gold standard of diagnostic test of *H. pylori* as well as the culture method. The accuracy of RUT is shown in Table 1.^104^, ^105^ In the Joint ESPGHAN / NASPGHAN guidelines, it is recommended that the initial diagnosis of *H. pylori* infection should be based on either positive histopathology (i.e. identification of organisms in microscopic evaluation of biopsies) and a positive RUT test or a positive culture. In consideration of a decline in the positive predictive value of the diagnostic tests due to decreasing infection rate, if there is inconsistency between histopathology and RUT, an additional urea breath test (UBT) or *H. pylori* stool antigen test (HpSA) is recommended.^3^

**Table 1:** Accuracy of each diagnostic test

Diagnostic test		Sensitivity	Specificity	Positive predictive value	Negative predictive value
Invasive tests	RUT^104^, ^105^	75–100%	84–100%	83–100%	94–96%
	Histological examination^104^	66–100%	94–100%	100%	96%
Non‐invasive tests	^13^C‐UBT (meta‐analysis)^107^	95.9% (95.3–96.4)	95.7% (95.3–96.0)	17.4 (14.6–20.7)^‡^	0.06 (0.05–0.07)^†^
	HpSA (meta‐analysis of ELISA method using monoclonal antibody)^109^	97.0% (94.0–98.0)	97.0% (95.0–98.0)	29.9 (10.3–86.9)^‡^	0.03 (0.02–0.07)^†^
	HpSA (meta‐analysis of IC methods using monoclonal antibody)^109^	88.0% (85.0–92.0)	93.0% (90.0–95.0)	10.6 (7.5–14.8)^‡^	0.11 (0.05–0.24)^†^
	Serum anti‐*H. pylori* IgG antibody test of EIA methods using JHM‐CAP in Japanese children^113^	93.0%	95.4%	90.9%	96.6%
	Serum anti‐*H. pylori* IgG antibody test of EIA methods in Japanese children^114^	91.2%	97.4%	35.6^‡^	0.09^†^
	Urine anti‐*H. pylori* IgG antibody test of ELISA methods in Japanese children^115^	94.4%	96.9%	94.4%	96.9%
	Urine anti‐*H. pylori* IgG antibody test of ELISA methods in Japanese junior high or high school students^116^	97.6%	96.5%	61.2%	99.9%
	Urine anti‐*H. pylori* IgG antibody test of IC methods in Japanese children^117^	78.4%	100%	100%	88.9%

EIA, enzyme immunoassay; ELISA, enzyme‐linked immunosorbent assay; HpSA, *Helicobacter pylori* stool antigen test; *H. pylori, Helicobacter pylori*; IC, immunochromatography; IgG, immunoglobulin G; JHM‐CAP, Japanese strain‐derived high‐molecular‐weight cell‐associated proteins; RUT, rapid urease test; UBT, urea breath test.

^†^ Negative likelihood ratio.

^‡^ Positive likelihood ratio.

**Statement 10‐1‐2: We recommend histological examination of gastric biopsies as a biopsy‐based diagnostic test for active *H. pylori* infection.**

Strength of recommendation: Weak. Evidence level: B. Agreement: 100%.

***Comments***

Histological examination allows the evaluation not only of *H. pylori* infection status but also the degree, phenotype, and distribution of gastritis. At least two biopsy specimens are obtained from the gastric antrum and the gastric corpus in clinical setting because the density of *H. pylori* colonization may be patchy and the severity of gastritis might be different depending on the anatomic location and the phenotype of the pathology associated with the infection.^3^, ^106^

**Statement 10‐1‐3: We recommend *H. pylori* culture because the culture method is the gold standard biopsy‐based test for active infection and it can also be used for antimicrobial susceptibility testing for optimization of eradication therapy.**

Strength of recommendation, Strong. Evidence level: Not applicable. Agreement: 100%.

***Comments***

Primary culture from gastric biopsies is the gold standard for the diagnosis of *H. pylori*.^3^ To perform antimicrobial susceptibility testing, positive culture is assumed. Gastric biopsy specimens for primary culture are recommended to be taken from the gastric antrum and the corpus, respectively.^3^

### B) Diagnostic tests without endoscopic biopsy specimens

**Statement 10‐2‐1: We recommend a ^13^C‐urea breath test as a diagnostic test for active *H. pylori* infection.**

Strength of recommendation: Strong. Evidence level: A. Agreement: 100%.

***Comments***

According to a recent meta‐analysis of the UBT in children,^107^ it is a reliable and accurate test to diagnose *H. pylori* infection (Table 1). A cutoff value of 3.5‰ is reported to be appropriate based on the study in Japanese children based on the receiver operating characteristic curve.^108^ In cases with near cutoff value, additional test is recommended.

**Statement 10‐2‐2: We recommend a stool antigen test as a diagnostic test for active *H. pylori* infection.**

Strength of recommendation: Strong. Evidence level: A. Agreement: 100%.

***Comments***

The HpSA is easy to perform and non‐invasive, and can be utilized even in infants and children who have difficulty collecting breath samples. In a meta‐analysis of HpSA in children,^109^ high accuracy, both sensitivity and specificity as well as positive and negative predictive values were reported from an enzyme‐linked immunosorbent assay (ELISA) using monoclonal antibodies (Table 1).

**Statement 10‐2‐3: We recommend against tests to detect anti‐** ***H. pylori* antibodies as single diagnostic tests in clinical settings to diagnose active *H. pylori* infection.**

Strength of recommendation: Strong. Evidence level: A. Agreement: 100%.

***Comments***

Anti‐*H. pylori* IgG antibodies using serum or urine samples are generally measured by ELISA or immunochromatography (IC). High false negative rates tests may occur for the antibody tests, particularly immediately following initial infection with *H. pylori*.^110^ On the other hand, antibodies to *H. pylori* can persist and continued to be positive for at least 1 year after therapy in 65% of the cohort of patients in the study who were successfully treated.^111^ The sensitivity of serum anti‐*H. pylori* IgG antibody testing in children under 10 years of age was reported to be as low as 51.4%.^112^ Recently, several studies reported that the sensitivity of antibody testing in the research setting is higher than conventional studies^113^, ^114^, ^115^, ^116^ (Table 1). However, the antibody tests do not directly determine presence of active *H. pylori* infection at the time of examination. Hence, antibody tests for *H. pylori* infection cannot be employed in the clinical setting due to the reasons for variability described and lack off appropriateness for the clinical setting. In addition, urine antibody tests may be even more variable than serological assays, and, may reveal high rates of false positives in cases of patients with proteinuria.^115^, ^116^, ^117^, ^118^ Based on the general performance characteristics of anti‐*H. pylori* antibody tests and the number of factors affecting their performance in different populations, the use of antibody test is not recommended in the clinical setting for the diagnosis of *H. pylori*, either as a single diagnostic test for *H. pylori* infection before eradication therapy. For example, there are numerous studies that demonstrate very low positive predictive values for the antibody test(s) when employed in the geographic areas of decreasing prevalence of *H. pylori* infection.

**[Clinical Question 11]**

**What do we need to do to increase the diagnostic accuracy for *H. pylori* infection?**

**Statement 11: We recommend more than two *H. pylori* tests such as two non‐invasive tests, i.e. breath test and stool test, or a biopsy‐based and non‐invasive test (i.e. breath test) for more accurate diagnosis of active infection.**

Strength of recommendation: Strong. Evidence level: C. Agreement: 100%.

***Comments***

It's recommended that the clinicians carry out more than two *H. pylori* tests for active infection with high accuracy to optimize the probability of *H. pylori* infection diagnosis. There is a report that an accuracy of diagnosis for *H. pylori* infection was enhanced when the results of HpSA and UBT became identical in children.^119^

**[Clinical Question 12]**

**What endoscopic findings are especially recommended for *H. pylori* tests (recommended in CQ10) for children who underwent upper gastrointestinal endoscopy for abdominal symptoms or anemia?**

**Statement 12: We recommend *H. pylori* tests when the following endoscopic findings are observed at diagnostic upper endoscopy: antrum‐predominant nodularity, ulcerations or erosions in the stomach or duodenum and / or disappearance of regular arrangement of collecting venules (RAC) in the gastric body.**

Strength of recommendation: Strong. Evidence level: C. Agreement: 100%.

***Comments***

To date, there have not been any systematic reviews or randomized controlled trials regarding CQ‐12. The sensitivity and specificity of endoscopically demonstrated nodular gastritis in the presence of active *H. pylori* infection were 44–98% and 100%, respectively.^17^, ^120^, ^121^, ^122^, ^123^ At the same time a confirmative diagnosis by biopsied specimens of gastric mucosa is recommended. The sensitivity and specificity of the endoscopic visualization of the regular arrangement of collecting venules pattern in the presence of active *H. pylori* infection are 96–100% and 87–88%, respectively.^124^, ^125^

**[Clinical Question 13]**

**When should we perform *H. pylori* testing to determine whether eradication of *H. pylori* was successful?**

**Statement 13: We recommend *H. pylori* testing for active infection four weeks or more after completion of eradication therapy to avoid false negative results.**

Strength of recommendation: Strong. Evidence level: C. Agreement: 100%.

***Comments***

In most clinical settings, either UBT or HpSA performed 4–8 weeks after eradication therapy has been associated with accurate performance.^126^, ^127^, ^128^, ^129^ The Joint ESPGHAN / NASPGHAN Guidelines for the Management of *Helicobacter pylori* in Children and Adolescents (Update 2016) recommend that the success of therapy should be monitored after 4‐8 weeks.^123^ Oderda *et al*. reported that both sensitivity and specificity were 100% in the examination two weeks after the completion of eradication therapy.^130^ It is expected to have clearer information after future cases have accumulated.

**[Clinical Question 14]**

**Which diagnostic test for *H. pylori* is recommended to determine whether eradication of *H. pylori* was successful?**

**Statement 14‐1: We recommend that the ^13^C‐urea breath test or stool antigen ELISA test using a monoclonal antibody be employed to confirm eradication**

Strength of recommendation: Strong. Evidence level: A. Agreement: 100%.

***Comments***

The sensitivity and specificity of each diagnostic test to confirm *H. pylori* eradication is shown in Table 2.^127^, ^128^, ^129^, ^130^, ^131^, ^132^, ^133^, ^134^, ^135^, ^136^, ^137^, ^138^, ^139^, ^140^, ^141^, ^142^, ^143^, ^144^, ^145^ Comparison with invasive diagnostic tests using endoscopic biopsy specimens (rapid urease test, histologic examination, and the culture method) showed the substantial accuracy of the UBT and the HpSA.^131^, ^132^, ^133^, ^134^, ^135^, ^136^, ^137^, ^138^ The UBT and HpSA were recommended to be used to determine whether *H. pylori* treatment was successful (i.e. as a test for cure) in several reviews^146^, ^147^, ^148^, ^149^, ^150^, ^151^, ^152^ and a meta‐analysis.^153^ The guidelines from ESPGHAN and NASPGHAN^3^ recommended the UBT or HpSA ELISA test using a monoclonal antibody to confirm eradication with high‐quality evidence. If the test results from the UBT or HpSA are in doubt, confirmation with another test result is preferable. Negative results from both UBT and HpSA are strong proof of the success of eradication therapy.

**Table 2:** Accuracy of each diagnostic test for confirmation of eradication treatment^†^

Diagnostic test		Sensitivity	Specificity	Positive predictive value	Negative predictive value
Non‐invasive methods	^13^C‐UBT^127^, ^131^, ^132^, ^133^, ^134^, ^135^	94.1–97.6%	92.3–98.8%	88.9–97.7%	96.0–98.8%
	^14^C‐UBT^136^	100%	100%	100%	100%
	HpSA ELISA test using a polyclonal antibody^129^, ^130^, ^131^, ^135^, ^137^, ^138^, ^139^, ^140^, ^141^, ^142^	86.7–100%	97.5–98.1%	86.7–91.7%	97.5–100%
	HpSA ELISA test using a monoclonal antibody^128^, ^129^, ^143^, ^144^, ^145^	95.6%	95.1–95.8%	86.3–88.0%	98.5–98.6%
	HpSA IC test using a monoclonal antibody^126^, ^136^, ^140^, ^143^, ^144^	60.0–75.0%	96.3–100%	77.8–100%	85.7–95.2%
	Serological tests using anti‐*H. pylori* IgG antibodies in serum^128^, ^135^	77.8%	32.1%	26.9%	81.8%
Invasive methods	Culture method^127^	70.6%	100%	100%	83.9%
	Histological examination^127^	100%	92.3%	89.5%	100%

^†^Data include Japanese manuscripts. ELISA, enzyme‐linked immunosorbent assay; HpSA, *Helicobacter pylori* stool antigen test; *H. pylori*, *Helicobacter pylori;* IC, immunochromatography; IgG, Immunoglobulin G; UBT, urea breath test.

**Statement 14‐2: We recommend against *H. pylori* tests using endoscopic biopsy specimens (rapid urease test, histological examination, and the culture method) to confirm the eradication of the infection.**

Strength of recommendation: Not determined. Evidence level: C. Agreement: 100%.

***Comments***

Non‐invasive methods (i.e. stool antigen test, UBT) can be employed as accurate “tests‐for‐cure” and thereby assess success or failure of eradication, so endoscopy and biopsy‐based tests are rarely needed – only to confirm eradication.^3^ Although the substantial accuracy of histologic examination was shown by Yañez *et al*.^127^ for confirmation of treatment (Table 2), invasive methods are not recommended in the current situation where accurate non‐invasive test can be performed.

**Statement 14‐3: We recommend against serological tests to detect anti‐ *H. pylori* antibodies as a single test to confirm eradication.**

Strength of recommendation: Strong. Evidence level: A. Agreement: 100%.

***Comments***

Serological tests using anti‐*H. pylori* IgG antibodies will be positive for months to years. Persistent positive results can lead to significantly decreased specificity^147^, ^148^, ^149^, ^150^, ^151^, ^152^ (Table 2). The ESPGHAN and NASPGHAN recommended against antibody‐based tests for *H. pylori* in the clinical setting^3^. On the other hand, serological assays using the amount of change of antibody titer as the confirmation method for treatment success / failure resulted in reasonably high sensitivity and specificity in several reports. The sensitivity and specificity were observed despite the variability of the serological assays from person to person, and the lack of quantitative value of antibody‐based tests. However, the decline in antibody titer in many patients took as long as 6–12 months and for these reasons, the usefulness of serological tests was felt to be much lower than that of other non‐invasive diagnostic tests available as tests for cure.^132^, ^152^

In conclusion, we suggest that serological tests using anti‐*H. pylori* antibodies should not be considered in the clinical setting for confirmation of *H. pylori* infection eradication therapy and should be considered only in special circumstances when UBT or HpSA would not be available for use.

## Treatment

**[Clinical Question 15]**

**What should the clinician take notice of for performing eradication therapies?**

**Statement 15‐1: In principle, we recommend eradication therapies for *H. pylori*‐infected children ≥ 5 years of age who have the diseases and / or clinical findings indicated for infection treatment.**

Strength of recommendation: Not applicable. Evidence level: D. Agreement: Not applicable.

**Statement 15‐2: We basically recommend employing a proton pump inhibitor‐based triple regimens including** ***H. pylori*‐susceptible antibiotics based upon antimicrobial susceptibility testing of the infecting organism, because of the increased clarithromycin‐resistant strains with rates of 40%–50%.**

Strength of recommendation: Strong. Evidence level: D. Agreement: 100%.

***Comments:*** common to Statements 15‐1 and ‐2.

Application of drug sensitivity testing for isolated *H. pylori* strains is most important to prevent not only eradication failure in first‐line therapy but also to try to prevent an increase in strains that are resistant to either clarithromycin (CAM) or metronidazole (MTZ). Resistance rates of *H. pylori* strains isolated from children to both CAM and MTZ have been increasing inside and outside Japan. In a 2014 report,^154^ it was shown that the overall resistance rate of *H. pylori* isolated from younger individuals (2–30 years old) in Japan was 57.9% (22 resistant strains / 38 strains tested).

The primary goal of eradication therapy is to obtain the highest possible eradication rate employing first‐line therapy. In the joint ESPGHAN/NASPGHAN guidelines, eradication therapy based on a drug sensitivity test for the isolated strains is recommended.^3^ This is a departure from the previous clinical practice guidelines. Now, *H. pylori* infection is finally being managed as an infectious disease and treated accordingly. When isolated strains are not CAM resistant, PPI + amoxicillin (AMPC) + CAM combination therapy is recommended. When the isolated strains are CAM resistant but not resistant to MTZ, PPI + AMPC+ MTZ combination therapy is recommended. When the isolated strains are resistant to both CAM and MTZ, PPI + AMPC (higher per kilogram dosing) + MTZ combination therapy is recommended. Clinical studies showed that tailoring therapy using a drug‐sensitivity test for the isolated strains before eradication therapy increased the eradication rate.^155^, ^156^, ^157^, ^158^, ^159^

**[Clinical Question 16]**

**Which eradication regimens are recommended as the first‐line therapies for *H. pylori* infection in children?**

**Statement 16‐1: We recommend a proton pump inhibitor‐based triple regimen with amoxicillin and clarithromycin (PAC regimen) if *H. pylori* strains are susceptible to clarithromycin or the antimicrobial susceptibility of the strains is unknown.**

Strength of recommendation: Strong. Evidence level: B. Agreement: 100%.

**Statement 16‐2: We recommend a proton pump inhibitor‐based triple regimen with amoxicillin and metronidazole (PAM regimen) as the first‐line therapy, if *H. pylori* strains are shown to be resistant to clarithromycin.**

Strength of recommendation: Strong. Evidence level: B. Agreement: 100%.

***Comments:*** common to Statements 16‐1 and −2.

The success rate of eradication therapy is attributed to two primary factors: antimicrobial resistance and adherence to medication.^2^, ^160^ CAM‐resistance is an important factor in failure of eradication therapies in children.^161^, ^162^ The CAM‐resistance rate is high in Japanese children, as shown in CQ15. Kato *et al*. reported that the primary eradication rate of PAC regimen in Japanese children was 91.7% for CAM‐sensitive strains.^161^ In another study from Japan, based on the result of an antibiotic susceptibility test, when the PAC regimen was used for CAM‐sensitive strains and PPI + AMPC + MTZ (the PAM regimen) for CAM‐resistant strains, the eradication rate was 93.4% in children and young adults.^154^ Based on these data, it is therefore recommended to use the regimen based on antimicrobial susceptibility test as a first‐line therapy – the PAC regimen if *H. pylori* strains are susceptible to the CAM or PAM regimen if isolate strains are CAM‐resistant. In a case of unknown antimicrobial susceptibility, it is recommended that the clinicians employ the PAC regimen because it is approved as the first‐line eradication therapy in Japan. Frequent prescriptions of MTZ raise concerns about creating additional resistance in other infectious diseases such as *Clostridioides* (formerly *Clostridium) difficile* or protozoa infections.

Regarding the duration of eradiation regimen in children, high‐quality large‐sized cohort studies comparing the various regimens remain limited.^160^ Based upon the approval of eradication therapy for Japanese adults, a 7‐day course of treatment regimen is basically recommended. However, if clinicians judge that there is a therapeutic need according to individual risk of eradication failure, then the eradication regimen should be employed as a longer duration regimen for up to 14 days.

We excluded *H. pylori* eradication regimens containing medications that are not approved in Japan, such as bismuth or sequential therapy, from the recommendation. The dosage of medications used for eradication therapy in children is shown in Table 3.^2^, ^161^, ^162^, ^163^, ^164^, ^165^, ^166^, ^167^

**Table 3:** Dosage of medications used for eradication therapy in children^2^, ^161^, ^162^, ^163^, ^164^, ^165^, ^166^, ^167^

	Dosage (mg/kg/day) Twice daily	Maximum daily dose (mg/day)
Proton pump inhibitors		
Lansoprazole	1.5	60
Omeprazole	1.0	40
Rabeprazole	0.5	20
Esomeprazole	≥4 years old Bodyweight < 30 kg, 20 mg/day Bodyweight ≥ 30 kg, 40 mg/day	40
Antibiotics		
Amoxicillin	50	1,500
Clarithromycin	15–20	800
Metronidazole	10–20	500

**[Clinical Question 17]**

**Which eradication regimens are recommended as the second‐line therapies in *H. pylori*‐infected children in whom the first‐line therapy failed?**

**Statement 17‐1: Eradication failure is most commonly due to poor drug adherence and/or *H. pylori* resistance to clarithromycin.**

Strength of recommendation: Not applicable. Evidence level: D. Agreement: Not applicable.

**Statement 17‐2: We recommend a proton pump inhibitor‐based triple regimen with amoxicillin and metronidazole (PAM regimen) for 7 days if *H. pylori* strains are resistant to clarithromycin.**

Strength of recommendation: Strong. Evidence level: D. Agreement: 100%.

***Comments***

The presence of CAM resistance is frequently attributed to *H. pylori* eradication failure. The PAM regimen was shown to be successful in children who failed in eradicating *H. pylori* with CAM containing triple therapy.^161^ However, the primary CAM resistance rate has risen to 40–60% in Japanese children.^154^, ^162^, ^168^ For children with CAM‐resistant *H. pylori,* the eradication rate with PAM regimen was reported as 100%^162^ and 94.3%.^160^ In patients with second‐line eradication failure, antimicrobial susceptibility should be obtained for the infecting *H. pylori* strain and salvage therapy should be tailored accordingly.^3^

**[Clinical Question 18]**

**What kinds of adverse effects associated with eradication therapy should be considered?**

**Statement 18: Adverse effects include but are not limited to skin eruption, diarrhea, loose stool, taste disturbance, dyspepsia, nausea, candidiasis, urticaria and hemorrhagic colitis as a severe symptom.**

Strength of recommendation: Not applicable. Evidence level: C. Agreement: Not applicable.

***Comments***

No systematic reviews or randomized controlled trials have been demonstrated against this CQ. In adults on PPI‐based eradication therapy, 6 to 34% of adverse effects were observed. No reports of severe adverse effects have been found in pediatric cases with PPI‐based eradication therapies.^159^, ^160^, ^161^, ^169^, ^170^, ^171^, ^172^, ^173^ Omeprazole‐based dual or triple regimens, despite being the most commonly used PPI, showed 33% of the adverse effects described. This should be taken into account when considering constituents of the triple therapy regimens.

**[Clinical Question 19]**

**Is a combination of probiotics with triple therapy eradication regimens effective for *H. pylori* treatment in children?**

**Statement 19: Improvement of the eradication rate by a combination of probiotics is not clear. However, it has been shown to be effective for the prevention of side effects including diarrhea.**

Strength of recommendation: Not applicable. Evidence level: C. Agreement: Not applicable.

***Comments***

When probiotics were combined with CAM‐based triple therapy in children, the eradication rate of *H. pylori* improved.^174^, ^175^ It was reported that the side effects as a whole, and each individual side‐effect such as diarrhea, nausea, vomiting, dyspepsia or dysphagia, which occurred with the conventional eradication therapy, significantly decreased by combining with probiotics (*P* < 0.05).^176^, ^177^, ^178^ However, the committee did not reach consensus on a recommendation because evidence about the probiotics was poor.

## Disclosure

S.F. has received speaker’s fees from Taisho Toyama Pharmaceutical Co., Ltd.

## Author contribution

All authors were involved in writing the manuscript and providing critical revision of the manuscript for important intellectual content. All authors read and approved the final manuscript.

## Funding information

The Japanese Society for Pediatric Gastroenterology, Hepatology and Nutrition provided the funding for the development of the present guidelines.
